# Robust Composites Based on Silicone Rubber for Self-Powered Piezoelectric Nanogenerators

**DOI:** 10.3390/polym17070977

**Published:** 2025-04-03

**Authors:** Vineet Kumar, Md Najib Alam, Siraj Azam, Sang Shin Park

**Affiliations:** School of Mechanical Engineering, Yeungnam University, 280 Daehak-ro, Gyeongsan 38541, Republic of Korea; vineetfri@gmail.com (V.K.); mdnajib.alam3@gmail.com (M.N.A.); sirajazam@gmail.com (S.A.)

**Keywords:** piezoelectric nanogenerators, silicone rubber, power density, mechanical stiffness, piezoelectric coefficients

## Abstract

Obtaining robust power density through piezoelectric nanogenerators (PENGs) is very challenging. Challenges include achieving good mechanical stability, optimum stiffness, reasonable voltage generation, limited heat dissipation, and power density as needed. This work focused exactly on these areas, and hybrid filler emerged as a promising candidate among the composites studied. For example, hybrid fillers exhibited optimized properties suitable for self-powered engineering applications. The composites fabricated in this work were based on titanium oxide (TiO_2_), molybdenum disulfide (MoS_2_), and silicone rubber (SR) as a host matrix. The results showed that TiO_2_ represents a good reinforcing filler, while MoS_2_ exerts a lubricating effect, improving the composites’ mechanical strength and elongation at break. For example, the compressive modulus at 8 per hundred parts of rubber (phr) was 2.39 MPa (TiO_2_), 1.62 MPa (MoS_2_), and 2.1 MPa (hybrid filler). Similarly, the hysteresis loss at 5 phr was 20.09 J/m (TiO_2_), 21.56 J/m (MoS_2_), and 20.48 J/m (hybrid filler). Moreover, the elongation at break at 8 phr was 150% (TiO_2_), 194% (MoS_2_), and 170% (hybrid filler). In the same way, the electro-mechanical properties obtained were also robust. For example, the voltage output was ~22 mV (TiO_2_), ~35 mV (MoS_2_), and ~46 mV (hybrid filler). Moreover, the PENGs developed in this work generated power. For example, the power density was ~0.55 pW/cm^2^ (TiO_2_), ~1.03 pW/cm^2^ (MoS_2_), and ~1.56 pW/cm^2^ (hybrid filler). Finally, the piezoelectric coefficient of the PENGs was 40 pC/N (TiO_2_), 112 pC/N (MoS_2_), and 160 pC/N (hybrid filler). These materials have a promising role in energy harvesting through self-powered nanogenerators for portable electronic systems. Finally, the low-power PENGs developed provide cost-effective voltage and power management circuits. This allows these PENGs to contribute to sustainable and self-sufficient electronic systems like pacemaker implants.

## 1. Introduction

Conventional batteries are widely used as portable power sources in small devices. However, they have various limitations such as finite lifespans, environmental concerns, and the need to be replaced or recharged [1]. These problems have fueled interest in alternative, self-sustaining energy-harvesting technologies. For example, PENGs are emerging as a promising substitute to these battery systems [2]. PENGs are portable devices that can produce power by converting mechanical energy into electrical energy. This power can be obtained by placing piezoelectric materials in an elastomeric matrix, inducing a “piezoelectric effect” [3]. Here, the “piezoelectric effect” refers to the generation of an electric charge when a material is subjected to mechanical stress or strain. By leveraging this phenomenon, PENGs can harvest the surrounding mechanical energy, such as vibrations, movements, or pressure. Then, they transform it into usable electrical energy like voltage or power density [4]. These advancements necessitate compact, sustainable, and reliable power sources that can operate independently and for extended durations. As stated, the working principle of PENGs is based on the coupling of mechanical deformation and electrical polarization in piezoelectric materials within an elastomer matrix [5]. Under mechanical deformations, the internal lattice of the piezoelectric material becomes distorted. This results in the separation of charges and the creation of an electric field. Finally, it induces a flow of electrons, which can be captured through electrodes and converted to an electrical output like voltage or portable power [6].

Traditionally used piezoelectric materials in PENGs include zinc oxide, lead zirconate titanate, and barium titanate. Moreover, environmentally friendly polymer-based materials like polyvinylidene fluoride or elastomers like SR are frequently used as matrices for PENGs [7,8]. There are various advantages to using PENGs as power sources and alternatives to traditional sources like batteries: (a) PENGs eliminate the need for external power sources by harvesting energy directly from the surrounding environment [9]. (b) Their nanoscale dimensions enable their integration into microelectronic devices with relatively small sizes or light weights. (c) PENGs offer a robust renewable and eco-friendly alternative to conventional power sources. The energy is harvested by utilizing ambient mechanical energy from the environment [10]. (d) PENGs can harvest energy from various sources, including human motion, environmental vibrations, and acoustic waves, and therefore, they can easily generate power. Finally, PENGs are versatile devices with no complex structure or chemical reactions [11]. Therefore, PENGs are highly durable, easy to use, and require minimal maintenance. So, they are cheap compared to battery systems that involve replacement or frequent maintenance. However, despite their many advantages, PENGs also suffer from some ongoing challenges [12]. These challenges include low power output and material stability, as well as the complexity of integration. Overall, scientists are expected to address these challenges and unlock the full potential of PENGs. For example, with advancements, as self-powered, sustainable energy sources, PENGs are expected to play a dominant role in the development of next-generation portable electronic devices [13].

An SR matrix is a synthetic elastomer that is composed of silicon, oxygen, and hydrogen. They are widely used in various applications due to their high thermal stability (−60 to 300 °C), and chemical resistance (ozone, water, etc.) [14]. Dielectric SR composites based on TiO_2_ and MoS_2_ offer a novel approach to developing flexible piezoelectric materials. Here, TiO_2_ enhances the dielectric properties and charge separation. Moreover, the MoS_2_ provides intrinsic piezoelectricity and flexibility. So, composites fabricated based on these materials exhibit robust electro-mechanical properties and are highly responsive materials [15]. These materials are profoundly useful for next-generation applications like wearable sensors and energy harvesters. Here, although SR does not inherently exhibit piezoelectricity, it serves as an excellent matrix for adding piezoelectric materials. After dispersing a piezoelectric material like barium titanate into SR, it can exhibit a piezoelectric response [16,17]. In the present work, TiO_2_ is selected as a material with high dielectric and energy applications. Moreover, even though TiO_2_ is not a strong piezoelectric material, it exhibits piezoelectricity in its anatase phase and nanoscale structure. Therefore, including TiO_2_ helps to achieve stronger dielectric properties, good interfacial polarization, and mechanical reinforcement. MoS_2_ is known to exhibit intrinsic piezoelectricity at the monolayer level due to its favorable symmetric structure. The role of MoS_2_ involves its intrinsic piezoelectricity, high charge carrier mobility, and high fracture toughness. These aspects make the hybrid filler fabricated in this work innovative for balanced properties. Overall, the use of TiO_2_ and MoS_2_ in an SR matrix as a composite material is useful to create advanced materials for specific applications. For example, TiO_2_ can provide reinforcement, while MoS_2_ contributes lubrication and mechanical strength. Moreover, MoS_2_ enhances multifunctional surfaces for self-cleaning, which can be useful for energy harvesting and storage or other advanced nanotechnology applications.

As described, there are various studies on PENGs in polymer composites [18,19,20,21]. However, most of these studies focus on energy harvesting with limited details. For example, these studies are limited to investigating mechanical properties or limited aspects of electro-mechanical properties like output voltage. Thus, to the best of the authors’ knowledge, there has been little attention paid to PENG functionality in terms of power density, charge density, and piezoelectric coefficients for SR-based composites. Moreover, composites based on hybrid fillers exhibit great properties due to synergism and novel routes of achieving higher levels of energy harvesting. For example, Yun et al. [18] published an interesting study on hybrid composites based on MXenes, MWCNTs, and PDMS. The results were promising, and a significantly higher voltage of up to 80 V was obtained. Moreover, a good power density of 13.8 W/m^2^ was reported. In another study, Chung et al. [19] developed robust composites based on carbon black and multiwall carbon nanotube hybrids. The results showed a relatively high output voltage of 0.5 V. Moreover, an elongation at a break of 1000% and an outstanding durability of 25,000 cycles were reported. In another study by Kumar et al. [19], MoS_2_ and diatomaceous earth hybrids were fabricated in SR, and improved properties were reported. For example, a stretchability of 140%, output voltage of 5.5 mV, and relatively high modulus of 6.1 MPa were reported. These values are quite promising for SR-based hybrid fillers in the literature.

However, these studies on hybrid fillers were limited to studying output voltage and did not report many other important energy parameters like power density, piezoelectric coefficient modulus, etc. [20]. Moreover, although great power density is reported in some studies, their shortcomings are that PVDF is non-stretchable, and the use of MXenes is very expensive. Keeping these points in mind, the present study aims to fill the literature gap regarding these energy-harvesting parameters and the use of cheap alternatives. For example, the present study explores materials and finds a power density of 1.56 pW/cm^2^, a charge density of 6.69 pC/cm^2^, and a piezoelectric coefficient of 160 pC/N. Moreover, the materials used in the present work are cheap, and the output voltage can be tuned by connecting the samples in series to obtain the voltage of choice. Overall, there are various advantages of the low-voltage PENGs developed in this work. For example, low-voltage PENGs can be useful for hearing aids and small audio devices that need low voltage to function. Moreover, energy harvesting through low-voltage PENGs can be useful for low-power devices like fitness trackers.

## 2. Experimental Section

### 2.1. Materials Used in This Work

The SR used as a matrix exhibits room-temperature vulcanization and has a commercial name of “KE-441-KT”. The vulcanizing agent used was a platinum-based catalyst with the commercial name “CAT-RM” following a condensation reaction. Both the rubber and the vulcanizing agent were obtained from Shin-Etsu Chemical Corporation, Tokyo, Japan. The TiO_2_ used has a 0-dimensional oval morphology and a particle size of 34 nm. TiO_2_ of this grade was purchased from PlasmaChem GmbH, Berlin, Germany. The MoS_2_ used has a flat structure with a 2-dimensional sheet-like morphology and particle size of ~2 µm. Moreover, MoS_2_ acts as a lubricating agent to ensure the high performance and durability of PENGs. It was purchased from Sigma-Aldrich, Saint Louis, MO, USA. Finally, the mold-releasing agent used was an anti-adhesive agent purchased from Nabakem, Pyeongtaek-si, Republic of Korea.

### 2.2. Characterization Section

Scanning electron microscopy (SEM) was used to study filler dispersion in the SR composites. The SEM instrument has the commercial name “S4800” and was purchased from Hitachi, Tokyo, Japan. After mechanical tests, the fractured composites were sectioned into 0.1 mm slices using a surgical blade for sample preparation. Then, the samples were mounted onto an SEM stub and subjected to platinum coating in a coating chamber for 2 min. Then, the samples were transferred to the SEM chamber for investigation. The mechanical properties were tested under compressive and tensile strain. First, a cylindrical sample with a 10 mm thickness and 20 mm diameter was subjected to 35% strain for compressive tests. The strain rate for these tests was 4 mm/min, load cell of 1 kN, and preload of 0.5 N. Dumbbell samples with a 2 mm thickness, gauge length of 4 mm, and width of 4 mm were used for testing mechanical properties under tensile strain. The strain rate for these tests was 200 mm/min, preload of 0.1 N, and load cell of 1 kN, tested according to standard DIN 53 504 [22]. The mechanical tests under cyclic compressive strain were performed on cylindrical samples at 30% cyclic strain. These tests were performed to study mechanical stability and to determine the amount of heat dissipated during the operation time. A universal testing machine (UTS) was employed for all mechanical tests and was purchased from Lloyd Instruments, Bognor Regis, UK. Finally, electrical mechanical tests like output voltage, and capacitance, were performed using both a UTS and real-time monitoring. The output voltage and capacitance were tested at 30% compressive cyclic strain. A multi-meter was used to record electrical properties of the composites during both UTS tests and real-time monitoring. A multi-meter with commercial name “34401A” was purchased from Agilent Technologies, Santa Clara, CA, USA. Finally, energy aspects such as power density and piezoelectric coefficient were calculated from the capacitance and output voltage. The piezoelectric coefficient was calculated from the generated charge per unit force applied in newtons.

### 2.3. Fabrication of Composites

The composites were prepared by mixing liquid silicone rubber with filler particles, as optimized in a previous study [21]. The filler particles used in composite fabrication were TiO_2_ and MoS_2_. The formulation is described in Table 1. The fabrication was initiated by placing a known amount of SR and filler particles in a beaker. Then, all the ingredients were mixed for 10 min until a uniform solution was achieved. Then, a known amount of vulcanizing agent was added to the composites and mixed for 1 min. Finally, the composites were poured into the molds. Then, these molds were kept for 24 h at room temperature for vulcanization. Finally, the samples are removed from the molds and tested for different properties and applications.

## 3. Results and Discussion

### 3.1. Filler Dispersion

**Figure 1:**
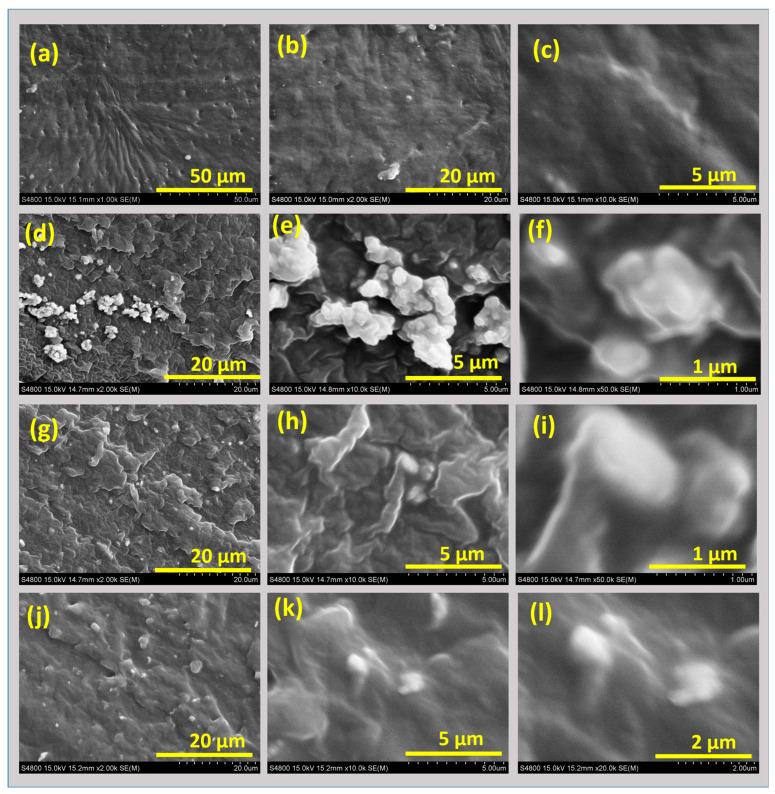
SEM images of different composites at 5 phr filler loading: (**a**–**c**) control sample; (**d**–**f**) TiO_2_-based samples; (**g**–**i**) MoS_2_-based samples; (**j**–**l**) hybrid samples.

It is well known that the dispersion of filler particles plays an important role in determining the output properties of rubber composites. For example, composites with uniform filler dispersion provide better properties than those with aggregates or agglomerates of filler particles [23]. Here, the filler dispersion of the composites was studied and is presented in Figure 1. Figure 1a–c shows the morphology of the unfilled or control sample at different resolutions. The micrographs show that the surface morphology of the SR matrix is rough. The SEM images further highlight the absence of cracks, voids, and phase separations. The results further suggest that certain holes are present and suppressed after adding fillers in the SR matrix.

Similarly, Figure 1d–f shows the effect of adding TiO_2_ to the SR matrix at different resolutions. The results show that in most cases, the TiO_2_ particles are uniformly distributed, as shown at low resolution. However, the high-resolution images show some partially aggregated particles that are expected to limit the overall performance of the composite. The high-resolution image also shows a good interface of TiO_2_ particles with the SR matrix. This evidence suggests better reinforcement of TiO_2_ with the SR matrix. This good interfacial strength also supports the good compatibility of TiO_2_ particles with the SR matrix [24]. Moreover, the smooth surface of the SR further proves good filler–rubber compatibility. These features provide support and lead to the high reinforcing capacity of these composites. The smooth surface also indicates the absence of crack formation, which indicates good stress distribution within the composite. Overall, controlling the filler content and fabrication method assists in achieving improved mechanical properties of TiO_2_ and are discussed in the following sections. In the same way, Figure 1g–i shows SEM images of MoS_2_ dispersion in the SR matrix at low and high resolutions. The micrographs show clear evidence of uniform dispersion of MoS_2_ at lower resolutions. However, some possible aggregation can be seen at higher resolutions. These features are due to van der Waals interactions among the MoS_2_ sheets and between adjacent sheets. However, this process is useful for the lubricating effect of the MoS_2_. For example, the lubricating effect helps in achieving higher dissipation losses, enabling their use for damping applications. Moreover, the lubricating effect promotes the smoothness of the SR matrix, thereby improving tensile strength and elongation at break. Therefore, the high-resolution SEM images show the effects of MoS_2_ on the smoothening of the SR matrix and suggest a good lubricating effect on the SR matrix. High-resolution SEM images also provide evidence for a good interface between MoS_2_ and the SR matrix. This further proves that the addition of MoS_2_ has good compatibility with the matrix, and thus, good properties are expected. Finally, Figure 1j–l shows the dispersion of hybrid fillers in the SR matrix. The low-resolution micrographs show that hybrid fillers have good dispersion in the SR matrix. Further high-resolution SEM images show the high affinity between TiO_2_ and MoS_2_ in the SR matrix. This behavior support possible synergism in the hybrid filler properties. As discussed already, both fillers show good interfacial interactions, and good compatibility is also indicated by the high-resolution SEM images. Therefore, the hybrid filler is expected to be better than using TiO_2_ or MoS_2_ as the only filler in the SR matrix. For example, the hybrid filler forms a unique heterogeneous filler networking. This result is a promising sign of optimum properties. For example, TiO_2_ provides stronger reinforcing properties, while MoS_2_ helps in exerting lubricating effects on the SR matrix. Thus, hybrid filler is expected to be superior and has optimum properties. These phenomena are justified and detailed in the following sections.

### 3.2. Mechanical Properties Under Static Compressive Strain

**Figure 2:**
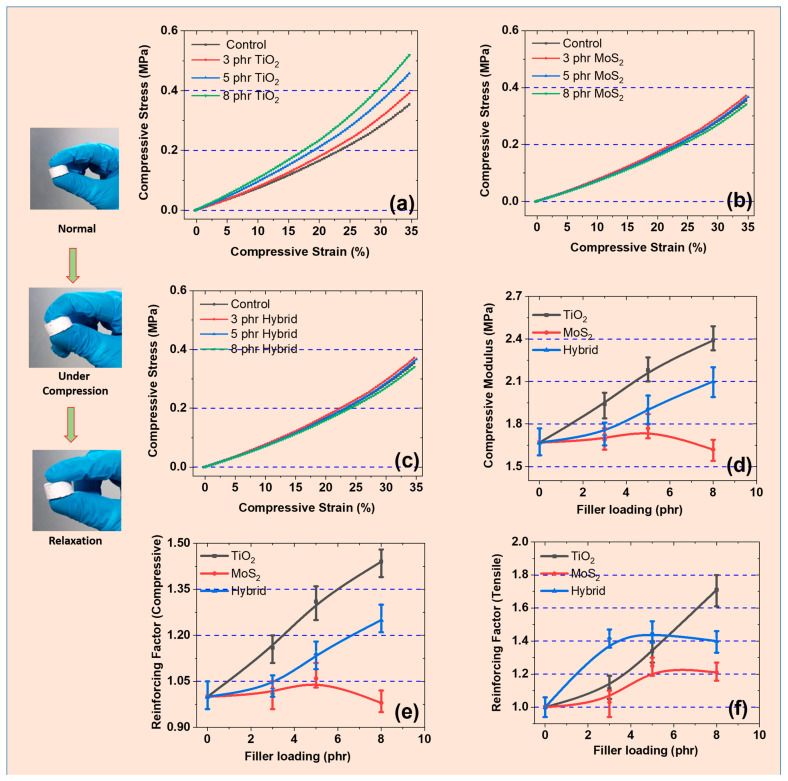
Mechanical properties of composites under compressive strain: (**a**–**c**) stress–strain curves for TiO_2_, MoS_2,_ and hybrid filler-based composites, (**d**) compressive moduli of the composites; (**e**) reinforcing factor under compressive strain, (**f**) reinforcing factor under tensile strain.

Mechanical properties are of the utmost importance when rubber composites are used in engineering applications like fatigue tests. These aspects are important for the use of such composites under constant mechanical load-like durability examinations [25,26]. Figure 2a–c shows the compressive stress–strain curves of different composites and their related compressive strength. The results demonstrate that when the cylindrical samples were subjected to mechanical strain, the compressive stress steadily increased, reaching a maximum at 35% strain. After this strain, the cylindrical samples cracked due to mechanical failure. The results further show that with the addition of fillers like TiO_2_, MoS_2_, or both, the compressive stress increases steadily. It was higher for TiO_2_ samples than for hybrid and MoS_2_-based samples. This higher stress could be attributed to a stronger compressive reinforcing effect of TiO_2_ on the SR matrix, as detailed in Figure 2e,f. These good reinforcing properties are also due to variations in particle size, surface chemistry, dispersion, and interactions with the SR matrix, as reported previously [27]. The mechanical properties were further tested to determine the compressive moduli of the composites, as shown in Figure 2d. As expected, the TiO_2_-based composites show an optimal mechanical performance, with higher moduli than other fillers. For example, the compressive modulus at 8 phr was 2.39 MPa (TiO_2_), 1.62 MPa (MoS_2_), and 2.1 MPa (hybrid filler). This was due to the small particle size (34 nm) of TiO_2_, which provides a higher interfacial area and better bonding of the TiO_2_ particles with the SR matrix [28]. SEM results further support these claims of strong filler–rubber interactions and good interfaces in these composites. In contrast, the MoS_2_-based composites have weak van der Waals interactions, leading to poor moduli. Moreover, the filler particles are distributed more efficiently, thereby reducing possible agglomerations, as supported by SEM images in Figure 1. However, the MoS_2_ tends to aggregate and agglomerate due to its layered structure and weak van der Waals interactions with the rubber matrix [29]. Moreover, the layered structure of MoS_2_ provides a lubricating effect that enhances wear resistance but not stiffness. Therefore, TiO_2_ is a preferred choice for applications demanding high strength, durability, and electro-mechanical stability in rubber composites.

Figure 2e shows the reinforcing factor under compressive strain. The results show that the reinforcing factor was higher for TiO_2_-, lower for MoS_2_-, and optimum for hybrid-filled composites. For example, the reinforcing factor at 8 phr was 1.44 (TiO_2_), 0.98 (MoS_2_), and 1.25 (hybrid filler). As stated, the higher reinforcing factor for TiO_2_ is due to better stiffness induced by TiO_2_ particles in the SR matrix. The other reasons include good interfacial bonding capability, which allows for better stress transfer and mechanical stability [30]. However, MoS_2_ has a lower reinforcing factor and is preferred to reduce friction and wear susceptibility. Similarly, hybrid fillers containing both TiO_2_ and MoS_2_ exhibit an optimum reinforcing factor. This shows that a hybrid-filled composite can be selected to obtain balanced properties. These properties include good stiffness, better fatigue resistance, and optimum electro-mechanical stability [31]. Similarly, Figure 2f shows the reinforcing properties under tensile strain. A synergistic effect of the hybrid filler was seen up to 5 phr loading before TiO_2_ domination at 8 phr. For example, at 5 phr, the reinforcing factor was 1.34 (TiO_2_), 1.25 (MoS_2_), and 1.45 (hybrid filler). This synergism is attributed to various factors. For example, MoS_2_ and TiO_2_ create a unique and more effective load transfer network within the SR matrix. This is also attributed to the TiO_2_-induced stiffness effect and the flexibility and better lubricating effect provided by MoS_2_ [32]. Their combination not only enhances the stress distribution but also reduces the weak points, thereby enhancing the tensile modulus. The synergism among the filler particles is also attributed to the improved compatibility. It is also due to the reduced agglomeration of hybrid fillers, thereby leading to better and more uniform reinforcement of the polymer matrix [15]. Moreover, the better physical interactions of TiO_2_ and the delamination or slippage induced by MoS_2_ in the SR matrix result in synergistic effects and thus a higher modulus in the hybrid composite.

### 3.3. Mechanical Properties Under Static Tensile Strain

**Figure 3:**
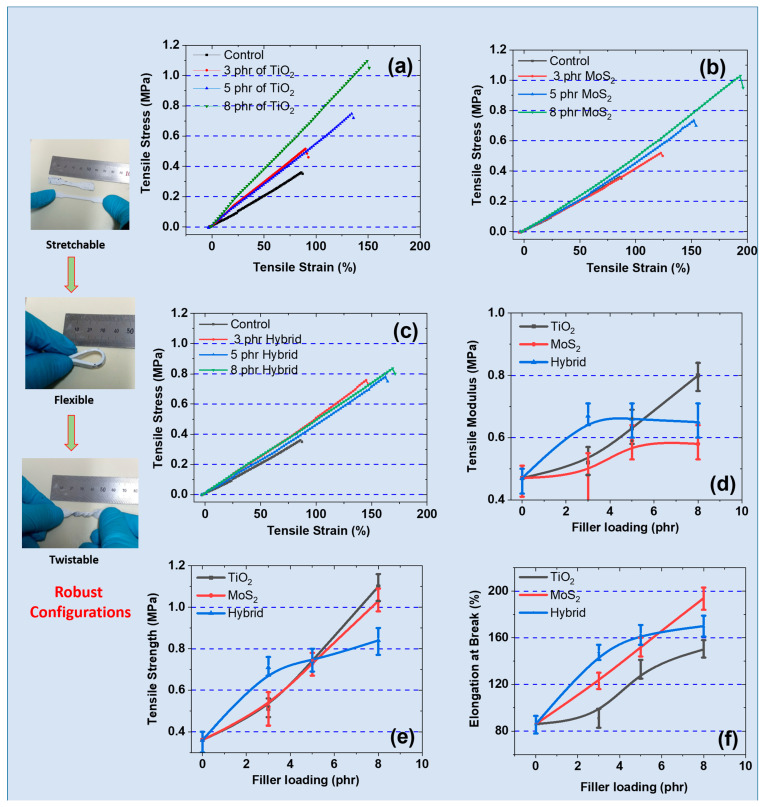
Mechanical properties of composites under tensile strain: (**a**–**c**) stress–strain curves of TiO_2_, MoS_2_, and hybrid-based composites; (**d**) tensile moduli of composites; (**e**) tensile strength of composites; (**f**) elongation at break of composites.

Generally, SR composites exhibit non-linear stress–strain behavior, where the stress increases with increasing strain. This behavior is proposed to occur because of molecular and structural transformation within the composite material under strain [33]. The static mechanical properties were further evaluated under tensile strain. Studying stress–strain is critical for engineering applications that require elasticity, durability, and high mechanical strength. Therefore, Figure 3a–c shows the stress–strain behavior of the composites under tensile strain. The results show higher stress under increasing strain until fracture. At low strain, these rubber composites exhibit elastic behavior, and the complete recovery of properties is witnessed, agreeing with the literature [34]. However, as the magnitude of strain increases to higher values, various reinforcing mechanisms come into existence. For example, the molecular chains of the SR matrix begin to stretch, and filler particles tend to align in the direction of the applied force. These features help in reducing entropy and increasing stiffness [35]. Moreover, the reinforcing effect of TiO_2_ and MoS_2_ enhances stress transfer within the composite sample, thereby increasing resistance to mechanical deformation.

Figure 3d shows the behavior of the tensile moduli of the rubber composites. As stated already, TiO_2_ is widely used as a reinforcing filler due to its favorable morphology and small particle size (34 nm). Moreover, the increasing reinforcement is also due to good compatibility with the SR matrix [36]. Similarly, MoS_2_ not only provides a reinforcing effect but also lubricating properties, making it useful for achieving high elongation at break and fatigue resistance. Therefore, when using these fillers in hybrid form, optimum and balanced properties as expected. These balanced properties include optimum stiffness, high fracture toughness, and better elongation at break [37]. Further, the results show that the tensile modulus was higher for hybrid samples up to 5 phr and then declined. For example, the tensile modulus at 5 phr was 0.63 ± 0.05 MPa (TiO_2_), 0.59 ± 0.04 MPa (MoS_2_), and 0.66 ± 0.06 MPa (hybrid filler). This could be attributed to synergism between TiO_2_ and MoS_2_ as hybrid components in the SR matrix [38]. Similarly, the tensile strength and elongation at break were obtained from the stress–strain curves and are presented in Figure 3e and Figure 3f, respectively. The results agree with the other mechanical properties observed. For example, the tensile strength and elongation at break are better for the MoS_2_-filled composite. Also, the tensile strength at 8 phr was 1.1 MPa (TiO_2_), 1.03 MPa (MoS_2_), and 0.84 MPa (hybrid filler).

These improved properties are due to the lubricating effects and favorable sheet-like morphology of these composites. These properties are attributed to better MoS_2_ networks and MoS_2_-SR interactions [39]. Moreover, the lubricating effect of MoS_2_ assists in reducing internal friction in MoS_2_-SR composites and thus improving elongation at break. For example, the elongation at break at 8 phr was 150% (TiO_2_), 194% (MoS_2_), and 170% (hybrid filler). However, up to 5 phr, the hybrid filler exhibited better tensile strength and elongation at break than the MoS_2_- and TiO_2_-only fillers. This is because of synergism among MoS_2_ and TiO_2_ as fillers, which results in achieving higher properties. For example, TiO_2_ provides better reinforcement, while MoS_2_ exhibits better elongation at break and tensile strength [40]. These balanced properties in hybrid fillers allow for improved durability and mechanical resilience in the composites. Therefore, these aspects make these hybrid-reinforced SR-based composites promising for high load-bearing and high-fatigue applications like energy harvesting.

### 3.4. Mechanical Properties Under Cyclic Compressive Strain

**Figure 4:**
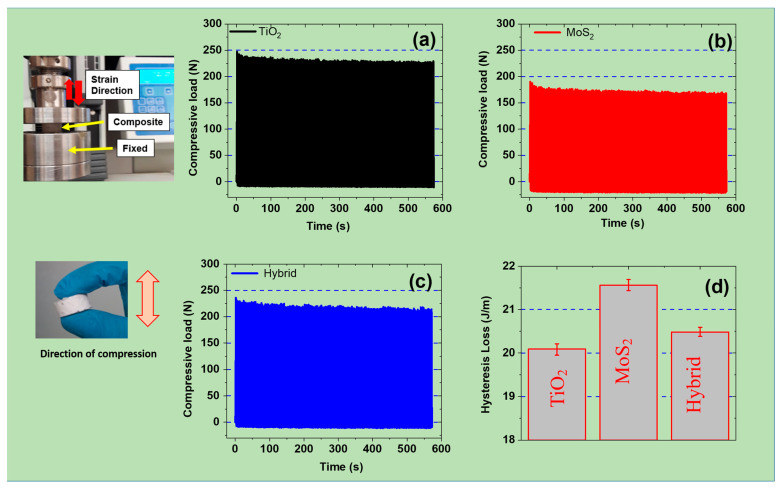
Mechanical properties of composites at 5 phr under a 30% compressive cyclic strain: (**a**–**c**) load vs. time curves for TiO_2_-, MoS_2_-, and hybrid-based composites; (**d**) hysteresis losses of the composites.

TiO_2_ and MoS_2_ fillers significantly influence mechanical properties like the durability and cyclic stability of SR composites. Here, the TiO_2_ enhances tensile strength and stiffness, while MoS_2_ provides lubrication and flexibility, as concluded in the previous sections. Moreover, it is also evident that the synergy between these fillers makes the composite well-suited for various engineering applications like energy harvesting. The high fatigue resistance, wear resistance, and elasticity under cyclic loading conditions are also important and thus studied [41]. Therefore, Figure 4a–c shows the mechanical stability of different composites under constant mechanical deformations. As expected, the TiO_2_-based samples show a higher compressive load compared to MoS_2_- and hybrid-filled composites. The improved mechanical properties are also due to the stronger reinforcing effect of TiO_2_ on SR-based composites. These results further show that the mechanical load was higher for initial cycles and stabilized in successive cycles. The higher compressive load during initial cycles was due to higher dissipation losses during these cycles [42]. However, this dissipation loss stabilized after successive cycles, and therefore, a stable phase was achieved. Moreover, MoS_2_- and hybrid-filled SR composites exhibited lower compressive loads. This was expected because of the weaker reinforcing effect of MoS_2_ on the SR matrix [43]. The other reason for the lower compressive load of MoS_2_-based composites is due to weak van der Waals interactions with the SR matrix. These results agree with the results achieved in Figure 2 and Figure 3 in the previous sections.

Similarly, the hysteresis losses of these composites were calculated from the initial and final compressive load curves under cyclic deformations (Figure 4d). Here, hysteresis loss refers to the energy dissipated as heat during the cyclic deformation of a material. The results show that the hysteresis losses at 5 phr were higher for MoS_2_ samples than TiO_2_- and hybrid-filled SR composites. For example, the hysteresis losses were 20.09 J/m (TiO_2_), 21.56 J/m (MoS_2_), and 20.48 J/m (hybrid filler). The higher hysteresis losses for MoS_2_-based SR composites were due to higher internal friction and energy dissipation. This favorable feature makes these materials more suitable for damping applications [44]. Moreover, the self-lubricating nature of MoS_2_ helps to reduce abrasive wear and fatigue damage. Similarly, TiO_2_ reduces hysteresis loss by restricting polymer chain mobility, resulting in lower energy dissipation per cycle. Moreover, TiO_2_ composites exhibit a high compressive load (elastic behavior), reducing damping effects [45]. Finally, the hybrid sample achieves a balance of mechanical stiffness and energy dissipation. Here, TiO_2_ contributes to structural stability and strength, while MoS_2_ enhances flexibility and damping ability. Therefore, the hybrid system modulates hysteresis loss, achieving a balance between energy efficiency and durability. This behavior makes these materials ideal for long-term fatigue operations during energy harvesting [46]. Overall, as per the needs of a given application, the ratio between TiO_2_ and MoS_2_ can be tuned. Therefore, these fillers enable customized energy dissipation, wear resistance, and mechanical resilience. This feature makes these SR-based composites suitable for long-term cyclic and dynamic loading conditions like mechanical stability during durability tests.

### 3.5. Electro-Mechanical Properties Under Cyclic Compressive Strain

**Figure 5:**
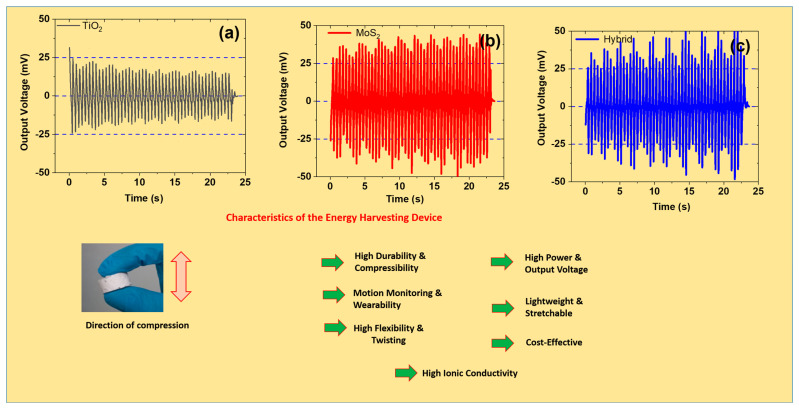
Electro-mechanical aspects of the composites at 5 phr under a 30% cyclic compressive strain: (**a**–**c**) output voltage vs. time for TiO_2_-, MoS_2_-, and hybrid-based composites.

SR is a robust matrix and is widely used in portable electronics, sensors, and other energy-harvesting applications. These features make it versatile due to its stretchability, easy processing, and dielectric properties [47]. It also has good intrinsic electro-mechanical performance that includes electric properties, dielectric properties, and electrical sensitivity to mechanical deformation [48]. However, these properties can be further enhanced by adding TiO_2_, and MoS_2_, and their hybrids to the SR matrix (Figure 5a–c). The output voltage was higher for MoS_2_- and hybrid-based composites than for those with TiO_2_ filler. For example, the voltage output was ~22 mV (TiO_2_), ~35 mV (MoS_2_), and ~46 mV (hybrid filler). TiO_2_ is known to exhibit a high dielectric constant and good compatibility with the SR matrix. Therefore, it results in improved charge storage capacity while maintaining stretchability and flexibility. Moreover, it improves the reinforcing properties that are required for electro-mechanical properties [49]. Similarly, the layered structure of MoS_2_ allows it to perform charge transfer and exhibit good response force under mechanical deformation. It also acts as a lubricating agent by reducing internal friction and results in good durability. In the same way, hybrid filler results in synergism by exhibiting higher dielectric properties, mechanical stiffness, and stability [50]. Therefore, a hybrid filler-based composite should be selected to obtain optimum balanced properties.

Great characteristics are exhibited by the energy-harvesting device developed in this work. The energy harvester exhibits all the necessary features that are required for multifunctional applications. For example, the device offers good durability for up to 1000 cycles and compressibility at up to 35% compressive strain. The good durability of the material can be used in harvesting energy for long-term operations without structural failure [51]. Moreover, it can recover after compression, thereby allowing it to harvest energy under constant dynamic loads. For example, a cylindrical sample can be compressed up to 35% and recover fully once the strain is removed. Moreover, it is a source of power density and promising output voltage. For example, the output voltage for PENG devices was ~22 mV (TiO_2_), ~35 mV (MoS_2_), and ~46 mV (hybrid filler). Moreover, calculating the power density is extremely important while studying the energy-harvesting aspects of the device. Hence, the PENGs developed in this work can generate power. For example, the power density was ~0.55 pW/cm^2^ (TiO_2_), ~1.03 pW/cm^2^ (MoS_2_), and ~1.56 pW/cm^2^ (hybrid filler). Moreover, wearable technology is a hot topic of research for flexible devices that are lightweight and offer good breathability for the skin. The present PENGs agree with all parameters of wearable devices and are thus useful for large-scale applications [52]. More importantly, the PENGs developed in this work are cost-effective and easily scalable. These features make them outstanding and versatile candidates for portable power sources. Overall, the developed PENGs are essential for designing next-generation multifunctional materials with high performance and reliability for energy sources.

### 3.6. Tuning of Stiffness and Output Voltage Under Cyclic Compressive Strain

**Figure 6:**
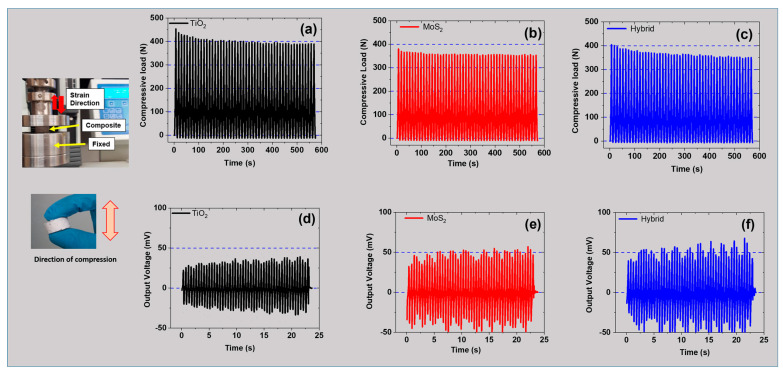
Properties at 5 phr under a 30% compressive cyclic strain: (**a**–**c**) compressive load vs. time for TiO_2_-, MoS_2_-, and hybrid-based composites; (**d**–**f**) output voltage vs. time for TiO_2_-, MoS_2_-, and hybrid-based composites.

The tuning of mechanical properties like stiffness and electro-mechanical properties like output voltage is very important for the desired applications. So, in this section, these properties are tuned by connecting two cylindrical samples in series and measuring their effect. Figure 6a–c shows the magnitude of compressive load for different composites. The results agree with the test of one sample in Figure 4. For example, the mechanical load was ~450 N for the TiO_2_ sample, ~400 N for the hybrid, and ~360 N for the MoS_2_. As discussed above, the higher mechanical load for TiO_2_-based composites is best for reinforcement. It is expected due to higher interfacial bonding between TiO_2_ and the SR matrix. This helps in achieving good filler dispersion and robust load transfer within the composite under mechanical strain [53]. Moreover, the TiO_2_-based composites have a higher modulus and stiffness than MoS_2_-based composites, which leads to better stress distribution in the composite. However, the MoS_2_ filler experiences weak van der Waals forces, leading to poor stress transfer within the composite. Moreover, MoS_2_ in hybrid fillers can create weak spots in the composite, thereby lowering the overall reinforcing effect, like in TiO_2_-based composites [54]. Overall, TiO_2_ exhibits better reinforcing properties, while MoS_2_ is beneficial for better lubricating properties, and their hybrid exhibits balanced properties.

Similarly, the electro-mechanical properties of these composites, including output voltage, were studied and are reported in Figure 6d–f. The results show that the MoS_2_-based composites provide better output voltage than TiO_2_ and hybrid filler systems. For example, the voltage output was ~40 mV (TiO_2_), ~50 mV (MoS_2_), and ~65 mV (hybrid filler). These results are in agreement with the results reported in Figure 5. As reported earlier, MoS_2_ has a semi-conductive nature and helps in improving the dielectric constant of the SR. Moreover, due to its layered structure, MoS_2_ contributes to intrinsic piezoelectricity within the structure [55]. TiO_2_ is not inherently piezoelectric, thereby making MoS_2_ a more favorable partner for electro-mechanical applications like sensors or PENGs. Moreover, the favorable spherical morphology of TiO_2_ helps in ensuring better dispersion compared to MoS_2_ with a layered structure. However, the hybrid filler system results in balanced conductive pathways and better mechanical stability than that achieved with TiO_2_ as the only filler. For example, the hybrid filler system can offer more balanced conductivity and mechanical stability [56]. Similarly, the hybrid system shows synergism by promoting higher electro-mechanical functionalities like output voltage and higher electrical properties with mechanical stability. Another benefit of using a hybrid filler is that MoS_2_ provides better flexibility, while TiO_2_ provides stiffness, thereby providing a better deformation-induced charge response [50]. Overall, MoS_2_- and hybrid-filled systems exhibit superior electro-mechanical properties compared to those with TiO_2_, which provides desirable stiffness in hybrid filler systems.

Over time, the advancement of material science and technology in terms of novel materials and novel processing techniques leads to the achievement of high performance for PENGs. For example, the output voltage evolved from a few millivolts to an order of magnitude higher and sometimes up to 100 V. These enhancements make PENGs more viable for real-world applications, including biomedical devices and the Internet of Things (IoT). However, current next-generation materials are either toxic, non-sustainable, expensive, or not useful for applications that require lower voltage. In contrast, low-voltage PENGs can be useful for power-implantable devices, bio-sensing, and drug delivery systems without requiring external batteries. Moreover, PENGs embedded in wound dressings can help monitor healing progress by supplying energy to micro-sensors. The other advantages of low-voltage PENGs are their use in wearable electronics, hearing aids, and small audio devices. These portable devices need a small voltage to function, and a higher voltage is not useful for such applications. Moreover, PENGs embedded in shoes, wristbands, or clothing can convert walking or hand movements into electrical energy for low-power devices like fitness trackers. Finally, low-voltage PENGs can be placed in bridges, buildings, and infrastructure to detect mechanical stress or vibrations, aiding in predictive maintenance and safety monitoring. The other advantages of the composites fabricated in the present study are their cost-effectiveness, sustainability, low toxicity, light weight, stretchability, flexibility, and durability. Therefore, these materials are useful for applications that require low voltage for functioning.

### 3.7. Capacitance Under Compressive Cyclic Strain

**Figure 7:**
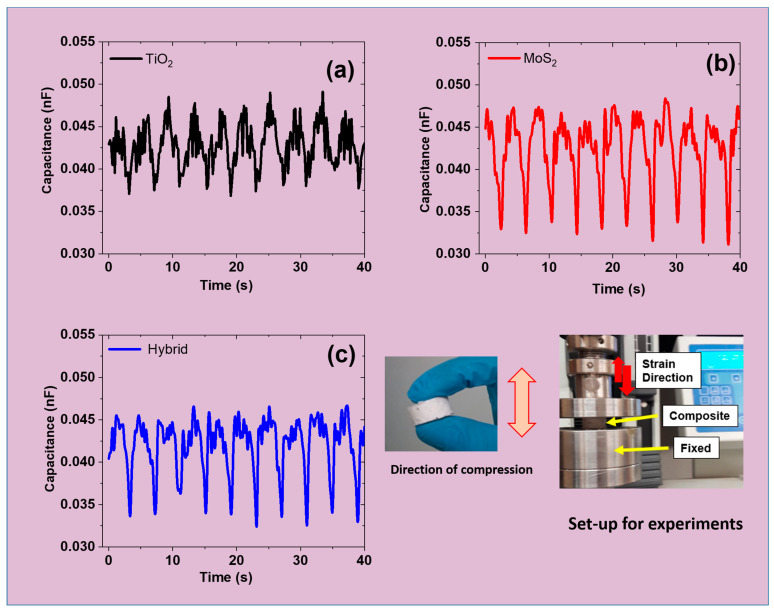
Capacitance of the different composites samples at 5 phr loading under a 30% compressive cycling strain: (**a**) TiO_2_ sample; (**b**) MoS_2_ sample; and (**c**) hybrid sample.

It is well known that capacitance plays a critical role in electro-mechanical properties. These electro-mechanical properties include the piezoelectricity or piezoresistivity of the composite material. Moreover, capacitance directly influences the energy-harvesting performance of PENG devices. These parameters include power density, charge density, and the piezoelectric coefficient [57]. The capacitance correlates with the dielectric constant, charge storage capacity, and polarization effect of the composite material. Therefore, the capacitance was studied in the present work for different composites based on TiO_2_, MoS_2_, and hybrid fillers in silicone rubber (Figure 7a–c). The addition of TiO_2_ can enhance charge storage and polarization responses. However, TiO_2_ acts as an insulating material, thereby limiting the overall charge transport [58]. In contrast, MoS_2_ has good charge transport properties due to its 2D sheet-like structure. Therefore, MoS_2_ improves the charge transport, thereby directly influencing the electrical response of the composite. Finally, the hybrid filler contributes to both capacitance and conductivity, making it more suitable for portable electronics systems like PENGs. Overall, the hybrid system achieves moderate capacitance with higher charge mobility [59]. It is also noteworthy that the hybrid filler system is expected to exhibit a synergistic effect, balancing the properties. These properties include the dielectric constant and charge transport, as detailed above. Moreover, the hybrid filler stimulates synergism, thereby being a capacitance booster, especially in devices like PENGs.

### 3.8. Energy Analysis and Piezoelectric Coefficients of PENGs

**Figure 8:**
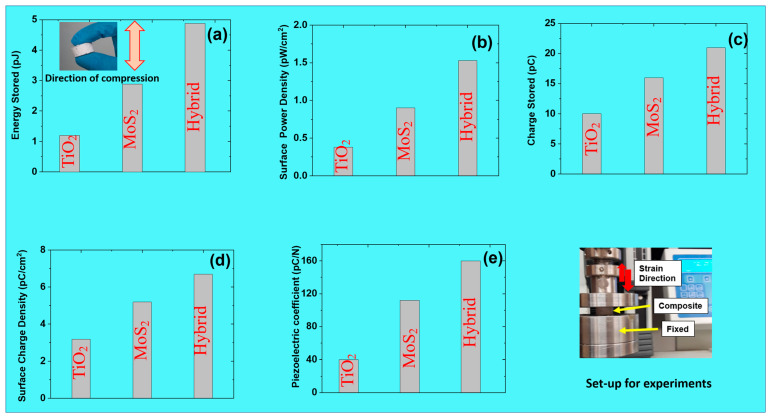
Energy-harvesting parameters of PENGs at 5 phr filler loading: (**a**) energy stored in composites; (**b**) surface power density of composites; (**c**) charge stored in composites; (**d**) surface charge density of composites; (**e**) piezoelectric coefficient of composites; and finally the set-up for obtaining capacitance used to calculate these parameters.

The use of PENGs as energy sources, especially for portable devices and remote areas, is continuously increasing nowadays. This is due to their easy-to-use, portable nature, with no need for charging stations like traditional batteries [60]. Here, energy parameters like energy stored, power density, charge density, and piezoelectric coefficient are studied (Figure 8a–d). All the energy parameters are derived from capacitance, as detailed in Figure 7. The results show that for almost all parameters, the energy output is better for the hybrid system than TiO_2_ or MoS_2_ as the only filler. For example, the surface power density was 0.38 pW/cm^2^ (TiO_2_), 0.90 pW/cm^2^ (MoS_2_), and finally 1.53 pW/cm^2^ (hybrid). This is due to charge transport and the accumulation of higher charge for the hybrid filler system compared to the TiO_2_- and MoS_2_-only filler systems. This charge transport mechanism favors higher energy output and current, thereby boosting the power density [61]. Moreover, the hybrid system provides better stress distribution, thereby improving mechanical stability. Similarly, the surface charge density was 3.18 pC/cm^2^ (TiO_2_), 5.09 pC/cm^2^ (MoS_2_), and finally 6.69 pC/cm^2^ (hybrid). Here, the hybrid filler provides improved strain-induced polarization in piezoelectric applications. However, the hybrid filler system creates a heterostructure, thereby increasing active surface area for charge accumulation [62]. Therefore, the high interfacial polarization creates better charge density for the hybrid filler system. These results also suggest that TiO_2_ and MoS_2_ may be synergized in a hybrid system. For example, TiO_2_ improves mechanical stability and reinforcement. Moreover, MoS_2_ improves fatigue properties, thereby balancing the hybrid system with good overall properties [63]. Therefore, this trend highlights that energy generation is best and most balanced for hybrid systems.

In addition, the piezoelectric coefficient is also important to determine the output energy and was thus studied, as shown in Figure 8e. For example, the piezoelectric coefficient of the PENGs was 40 pC/N (TiO_2_), 112 pC/N (MoS_2_), and 160 pC/N (hybrid filler). Thus, the results show that values were higher for hybrid filler and in agreement with the energy parameters. There is great value in studying energy parameters, including the piezoelectric coefficient [64]. For example, these studies help in understanding optimal mechanical-to-electrical energy conversion. Moreover, by optimizing the piezoelectric coefficient, these composite materials can be useful for pressure, strain, and human motion sensing [65]. Additionally, a well-characterized piezoelectric coefficient helps design efficient and responsive wearable technologies. Finally, the piezoelectric coefficient allows researchers to optimize the trade-off between flexibility and energy output. Overall, by optimizing the piezoelectric response, one can improve efficiency and durability. Moreover, the mechanical adaptability paves the way for next-generation smart materials [66].

### 3.9. Real-Time Monitoring of PENGs

**Figure 9:**
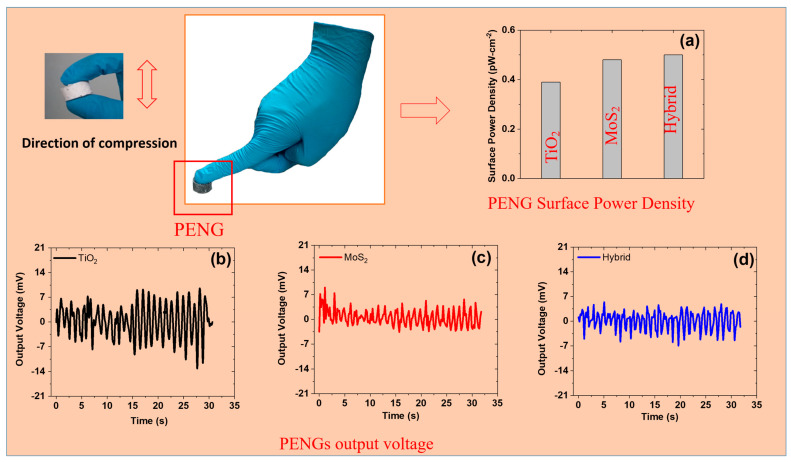
Real-time monitoring of PENGs through finger pressing at 5 phr filler loading: (**a**) surface power density; (**b**–**d**) output voltage of TiO_2_, MoS_2_, and hybrid filler system composites.

In addition to measuring power density in the lab using the UTS machine, it is important to achieve this power in real time. Here, we demonstrate power generation through human motions like finger pressing of the PENG devices (Figure 9). As detailed above, the power density was best for the hybrid filler system, compared to TiO_2_ and MoS_2_ single-filler systems (Figure 9a). For example, the surface power density was ~0.39 ± 0.03 pW/cm^2^ (TiO_2_), ~0.48 ± 0.04 pW/cm^2^ (MoS_2_), and ~0.5 ± 0.04 pW/cm^2^ (hybrid filler). Thus, these results agree with the above results obtained through universal testing in lab conditions. Here, the hybrid fillers exhibit synergism and enhance the piezoelectric strain response. Moreover, they also provide a stronger polarization effect in composite materials [67]. Therefore, higher energy output can be achieved under human motions like finger pressing compared to that achieved with TiO_2_ or MoS_2_ as a single filler. Moreover, the finger pressing of the hybrid composite allows for more efficient energy generation under constant pressing of the PENG device. This helps in achieving consistent power even at low strain or with irregular human motions [68]. Also, the hybrid filler system enhances piezoelectric charge transfer, thereby leading to higher power density compared to systems with TiO_2_ or MoS_2_ as a single filler.

The output voltage generated by finger pressing is presented in Figure 9b–d. The results show that the output voltage was similar for all composites in real-time monitoring. For example, the voltage output was ~7.7 mV (TiO_2_), ~8.3 mV (MoS_2_), and ~6.9 mV (hybrid filler). Here, the trend is in fair agreement with the results obtained using the universal testing machine in the lab. This behavior is attributed to the limited mechanical force of 1–5 N provided by the pressing. This force is much lower than the force applied through the UTS machine in the lab. So, as the piezoelectric effect is dependent on applied strain, the lower force by finger pressing may not provide a high output voltage [69]. Therefore, the force is too small to make a difference in the results from machine testing. Moreover, the voltage generated by the piezoelectric effect is dependent on stress-induced polarization [70]. For example, low force results in lower charge generation that may not be significantly different between TiO_2_-, MoS_2_-, and hybrid-filled systems. Additionally, as finger pressing involves limited mechanical force, charge transfer efficiency remains similar across all fillers studied in the polymer rubber matrix [71]. Overall, the hybrid filler systems benefit from both TiO_2_ and MoS_2_, but at low forces, they do not show a large difference in output voltage. Finally, hybrid fillers often outperform TiO_2_ and MoS_2_ single-filler systems in power density due to good charge transfer and better mechanical response. The results described in this work can be useful for various real applications. For example, the power generated in this work can be useful for generating voltage while walking. Furthermore, the PENGs developed in this work can be useful as implants for pacemakers, thereby eliminating the need for a battery. Moreover, these PENGs can be installed on bridges and buildings to harvest energy from vibrations. This energy can be useful to monitor structural health without requiring external power sources. Additionally, the PENGs developed in this work can be integrated into mechanical keyboards to generate power from keystrokes. Finally, real-time monitoring is demonstrated in Figure 9, showing the power produced through finger pressing of the PENG system. Thus, this work is useful for generating sustainable and limitless power sources for small power devices.

### 3.10. Mechanical and Electro-Mechanical Durability Tests

**Figure 10:**
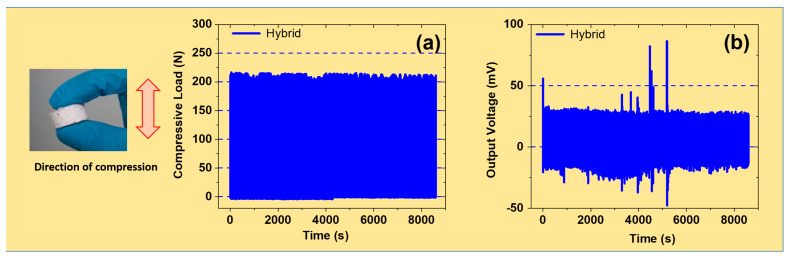
Durability of hybrid fillers under compressive cyclic mechanical deformations for 3000 cycles: (**a**) durability tests for mechanical properties; (**b**) durability tests for electro-mechanical properties.

The durability of a composite under cyclic compressive strain is an important factor for its use in energy-harvesting-related applications. To enhance the durability and load-bearing mechanical properties, reinforcing fillers are often added [72]. Here, TiO_2_ and MoS_2_ were added to the SR matrix as hybrid fillers. TiO_2_ was added to improve reinforcing properties, while MoS_2_ was added to improve tribological properties. Figure 10a shows that the compressive load was stable for up to 3000 cycles without any mechanical failure. This shows that the hybrid filler system developed in this work has excellent prospects for long-term operation. As described earlier, TiO_2_ and MoS_2_ act as an optimum and balanced filler for obtaining great durability characteristics. Various factors affect durability, such as filler dispersion, filler content, and environmental factors like humidity [73]. Here, the filler dispersion was optimum for the 5 phr hybrid filler, as justified in Figure 1, which thus supports stable durability. Furthermore, the filler content was optimum, as it was free from large aggregates and agglomerates, and thus supports stable durability. Finally, stable and optimum environmental conditions like humidity promote good durability features. Therefore, the hybrid system developed in this work provides balanced and stable durability. Moreover, it represents a promising candidate for use on an industrial scale.

Regarding PENGs’ durability prospects, TiO_2_ and MoS_2_ hybrid fillers in SR significantly improved durability and exhibited long-term functionality. Figure 10b shows the electro-mechanical durability behavior of the hybrid-filled composites based on an SR matrix. The results show that the voltage drop was negligible for up to 3000 cycles under cyclic mechanical strain. Moreover, the results show that hybrid fillers were developed to offer improved durability and functional efficiency in piezoelectric applications. Here, the TiO_2_ component enhances charge trapping and polarization, improving piezoelectric output and long-term stability. Moreover, MoS_2_ aids in charge mobility and contributes to overall enhanced electro-mechanical coupling. Therefore, both fillers exhibit great functionalities that result in good and synergistic piezoelectric output voltage. There are various prospective applications for PENGs developed through hybrid fillers. These applications include wearable electronics, structural health monitoring, and IoT wireless systems [74]. Wearable electronics benefit from durability and flexible energy harvesters for different sensors. Additionally, health monitoring is assisted by self-powered sensors for detecting mechanical strains and electro-mechanical failures. Finally, IoT systems are supported by reliable and long-lasting nanogenerators for low-power devices.

## 4. Conclusions

The addition of TiO_2_ and MoS_2_ in both single and hybrid states to an SR matrix resulted in robust PENGs for energy harvesting. This study shows that TiO_2_ can act as a good reinforcing filler and improve the mechanical stability of the composites. This will help in obtaining improved stiffness and is therefore suited for load-bearing applications. Moreover, MoS_2_ acts as a lubricant, thereby improving the composites’ tensile strength and elongation at break. These lubricating properties and favorable 2D sheet-like morphology are suitable for applications requiring high durability and stable electrical mechanical features. The higher compressive modulus of TiO_2_ supported its ability to improve mechanical stiffness, while optimum stiffness was achieved for the hybrid filler system. Here, the favorable morphology and lubricating effect of MoS_2_ help in achieving higher dissipation losses that are useful for damping applications. Moreover, the elongation at break at 8 phr was 150% (TiO_2_), 194% (MoS_2_), and 170% (hybrid filler). A higher elongation at break was achieved with MoS_2_-filled composites due to the lubricating effect of MoS_2_ on SR composites. In the same way, the electro-mechanical properties obtained were also robust. Here, as expected, synergism was also seen in power density, which makes the hybrid filler promising as a power source. Finally, the piezoelectric coefficient of the PENGs was 40 pC/N (TiO_2_), 112 pC/N (MoS_2_), and 160 pC/N (hybrid filler). Overall, as proposed in the abstract, the hybrid filler shows synergism, and promising balanced properties are found. For example, hybrid-filled composites exhibit optimum stiffness (2.1. MPa), compared to 2.39 MPa (TiO_2_) and 1.62 MPa (MoS_2_) for single-filler systems. Moreover, the hybrid filler exhibits a reasonable output voltage. For example, the voltage output was ~22 mV (TiO_2_), ~35 mV (MoS_2_), and ~46 mV (hybrid filler). Here, the hybrid system promotes synergism, and a higher voltage is generated compared to that for MoS_2_ or TiO_2_ as the only filler. Furthermore, the hybrid filler exhibits controlled heat dissipation. For example, the hysteresis loss was 20.09 J/m (TiO_2_), 21.56 J/m (MoS_2_), and optimum for the hybrid filler (~20.48 J/m). Moreover, the hybrid filler exhibits better energy generation prospects. For example, the power density was ~0.55 pW/cm^2^ (TiO_2_), ~1.03 pW/cm^2^ (MoS_2_), and ~1.56 pW/cm^2^ (hybrid filler). Finally, the hybrid filler exhibits better mechanical stability under compressive strain than MoS_2_ or TiO_2_ as the only filler. Its mechanical stability can be evidenced in Figure 4, Figure 5 and Figure 6. Moreover, the durability tests for 3000 cycles in Figure 10 further support good mechanical and electro-mechanical properties.

## Figures and Tables

**Table 1:** Formulation table: all amounts in per hundred parts of rubber (phr).

Sample Details	Amount of Silicone Rubber	Amount of TiO_2_	Amount of MoS_2_	Amount of Vulcanization Agent
Control	100	-	-	2
TiO_2_ Composite	100	3, 5, 8	-	2
MoS_2_ Composite	100	-	3, 5, 8	2
Hybrid Composite	100	1.5, 2.5, 4	1.5, 2.5, 4	2

## Data Availability

The original contributions presented in this study are included in the article. Further inquiries can be directed to the corresponding authors.

## References

[1] Olabi A.G., Abbas Q., Shinde P.A., Abdelkareem M.A. (2023). Rechargeable batteries: Technological advancement, challenges, current and emerging applications. Energy.

[2] Hu D., Yao M., Fan Y., Ma C., Fan M., Liu M. (2019). Strategies to achieve high performance piezoelectric nanogenerators. Nano Energy.

[3] Owusu F., Venkatesan T.R., Nüesch F.A., Negri R.M., Opris D.M. (2023). How to Make Elastomers Piezoelectric?. Adv. Mater. Technol..

[4] Wu M., Zhu C., Liu X., Wang H., Si J., Xu M., Mi J. (2024). Recent advances in nanogenerators driven by flow-induced vibrations for harvesting energy. Mater. Today Energy.

[5] Dong K., Peng X., Wang Z.L. (2020). Fiber/fabric-based piezoelectric and triboelectric nanogenerators for flexible/stretchable and wearable electronics and artificial intelligence. Adv. Mater..

[6] Kumar V., Manikkavel A., Yewale M.A., Alam M.N., Park S.S. (2024). Lightweight, compressible, stretchable, ultra-soft, and mechanically stable composites for piezo-electric energy generators and strain sensing. Mater. Res. Bull..

[7] Bhadwal N., Ben Mrad R., Behdinan K. (2023). Review of zinc oxide piezoelectric nanogenerators: Piezoelectric properties, composite structures and power output. Sensors.

[8] Hu S., Shi Z., Zhao W., Wang L., Yang G. (2019). Multifunctional piezoelectric elastomer composites for smart biomedical or wearable electronics. Compos. Part B Eng..

[9] Hu Y., Wang Z.L. (2015). Recent progress in piezoelectric nanogenerators as a sustainable power source in self-powered systems and active sensors. Nano Energy.

[10] Singh P.K., Kaur G.A., Shandilya M., Rana P., Rai R., Mishra Y.K., Tiwari A. (2023). Trends in piezoelectric nanomaterials towards green energy scavenging nanodevices. Mater. Today Sustain..

[11] Nguyen Q.H., Ta QT H., Tran N. (2022). Review on the transformation of biomechanical energy to green energy using triboelectric and piezoelectric based smart materials. J. Clean. Prod..

[12] Hu Y., Lin L., Zhang Y., Wang Z.L. (2012). Replacing a battery by a nanogenerator with 20 V output. Adv. Mater..

[13] Zhao Z., Dai Y., Dou S.X., Liang J. (2021). Flexible nanogenerators for wearable electronic applications based on piezoelectric materials. Mater. Today Energy.

[14] Kumar V., Alam M.N., Manikkavel A., Song M., Lee D.J., Park S.S. (2021). Silicone rubber composites reinforced by carbon nanofillers and their hybrids for various applications: A review. Polymers.

[15] Bhatt A., Singh V., Bamola P., Aswal D., Rawat S., Rana S., Sharma H. (2023). Enhanced piezoelectric response using TiO2/MoS2 heterostructure nanofillers in PVDF based nanogenerators. J. Alloys Compd..

[16] Möls K., Aarik L., Mändar H., Kasikov A., Niilisk A., Rammula R., Aarik J. (2019). Influence of phase composition on optical properties of TiO_2_: Dependence of refractive index and band gap on formation of TiO_2_-II phase in thin films. Opt. Mater..

[17] Latief U., Mondal S., Aishwarya A., Bhattacharjee N., Bhattacharyya A.R. (2025). Hybrid Fillers of Barium Titanate and Functionalized Multi-Walled Carbon Nanotubes Incorporated Poly (vinylidene fluoride) Nanocomposites for Piezoelectric Nanogenerators. ACS Appl. Nano Mater..

[18] Yun J., Park J., Ryoo M., Kitchamsetti N., Goh T.S., Kim D. (2023). Piezo-triboelectric hybridized nanogenerator embedding MXene based bifunctional conductive filler in polymer matrix for boosting electrical power. Nano Energy.

[19] Chung K.Y., Xu B., Li Z., Liu Y., Han J. (2023). Bioinspired ultra-stretchable dual-carbon conductive functional polymer fiber materials for health monitoring, energy harvesting and self-powered sensing. Chem. Eng. J..

[20] Kumar V., Alam M.N., Yewale M.A., Lee D.J., Park S.S. (2024). Mimicking self-powered piezoelectric energy-generating behavior in silicone rubber composites under compressive and tensile strains. ACS Appl. Electron. Mater..

[21] Kumar V., Sood A., Kumar A., Yewale M.A., Alam M.N., Park S.S. (2025). Modulating the energy harvesting with tunable hardness from mildly functionalized graphite nanoplatelets-based composites for wearable applications. J. Alloys Compd..

[22] (2017). Testing of Rubber—Determination of Tensile Strength at Break, Tensile Stress at Yield, Elongation at Break and Stress Values in a Tensile Test.

[23] Lee G., Jang H.G., Cho S.Y., Joh H.I., Lee D.C., Kim J., Lee S. (2024). Polyethylene-derived high-yield carbon material for upcycling plastic wastes as a high-performance composite filler. Compos. Part C Open Access.

[24] Yang T., Wang C., Liu L., Zhang L. (2024). Silicone elastomer dielectric composites by introducing novel O-MMT@ TiO_2_ nanoparticles for energy harvesting application. Compos. Part A Appl. Sci. Manuf..

[25] Jiang S., Yong Z. (2024). Modulation of Mechanical Properties of Silica-Filled Silicone Rubber by Cross-Linked Network Structure. Polymers.

[26] Mobtasem M., Abd-Elhady A.A., Sallam H.E.D.M. (2024). Implementation of a new approach based on the functionally graded materials concept to improve the strength of laminated composites containing open-hole. Polym. Compos..

[27] Zeng Y., Xiong C., Li J., Huang Z., Du G., Fan Z., Chen N. (2021). Structural, dielectric and mechanical behaviors of (La, Nb) Co-doped TiO_2_/Silicone rubber composites. Ceram. Int..

[28] Liu J., Yao Y., Chen S., Li X., Zhang Z. (2021). A new nanoparticle-reinforced silicone rubber composite integrating high strength and strong adhesion. Compos. Part A Appl. Sci. Manuf..

[29] Yang S., Li R., Zhu H., Zhang F., Yang X., Tan Q., Du J. (2025). Fabrication of an Environmentally Friendly Modifier Based on DAh-MT-MoS_2_ via Codeposition for the Preparation of Composite TB Rubberized Asphalt. J. Mater. Civ. Eng..

[30] Kumar V., Kumar A., Song M., Lee D.J., Han S.S., Park S.S. (2021). Properties of silicone rubber-based composites reinforced with few-layer graphene and iron oxide or titanium dioxide. Polymers.

[31] Borgaonkar A.V., Ismail S. (2022). Tribological behavior prediction of composite MoS_2_-TiO_2_ coating using Taguchi coupled artificial neural network approach. Proc. Inst. Mech. Eng. 2022 Part C J. Mech. Eng. Sci..

[32] Sethurajaperumal A., Srivastava S., Ganesh G., Sundara R., Varrla E. (2024). Natural surfactant stabilized aqueous MoS_2_ nano-lubricants for reducing friction and wear. Chem. Eng. J..

[33] Kumar V., Alam M.N., Park S.S. (2024). Review of Recent Progress on Silicone Rubber Composites for Multifunctional Sensor Systems. Polymers.

[34] Leng D.X., Huang C., Xu K., Ma Y., Liu G.J., Li Z.X. (2021). Experimental mechanics and numerical prediction on stress relaxation and unrecoverable damage characteristics of rubber materials. Polym. Test..

[35] Mogbojuri G., Abtahi S., Hendeniya N., Chang B. (2025). The Effects of Chain Conformation and Nanostructure on the Dielectric Properties of Polymers. Materials.

[36] Nawaz H., Umar M., Maryam R., Nawaz I., Razzaq H., Malik T., Liu X. (2022). Polymer Nanocomposites based on TiO_2_ as a reinforcing agent: An Overview. Adv. Eng. Mater..

[37] Shirvanimoghaddam K., Balaji K.V., Yadav R., Zabihi O., Ahmadi M., Adetunji P., Naebe M. (2021). Balancing the toughness and strength in polypropylene composites. Compos. Part B Eng..

[38] Kumar V., Alam M.N., Yewale M.A., Park S.S. (2024). Multifunctional Aspects of Mechanical and Electromechanical Properties of Composites Based on Silicone Rubber for Piezoelectric Energy Harvesting Systems. Polymers.

[39] Qian C., Chen J., Wang S., Wang M., Song S. (2024). Molecular dynamics investigation on the thermal-oxidative aging and mechanical properties of nitrile butadiene rubber composites with molybdenum disulfide. Appl. Phys. A.

[40] Chen Z., Zhang M., Guo Z., Chen H., Yan H., Ren F., Ren P. (2023). Synergistic effect of novel hyperbranched polysiloxane and Ti_3_C_2_T_x_ MXene/MoS_2_ hybrid filler towards desirable mechanical and tribological performance of bismaleimide composites. Compos. Part B Eng..

[41] Zhai W., Bai L., Zhou R., Fan X., Kang G., Liu Y., Zhou K. (2021). Recent progress on wear-resistant materials: Designs, properties, and applications. Adv. Sci..

[42] Persson A.M.M.R., Andreassen E. (2022). Cyclic compression testing of three elastomer types—A thermoplastic vulcanizate elastomer, a liquid silicone rubber and two ethylene-propylene-diene rubbers. Polymers.

[43] Zeng Y., Tang L. (2023). Improved dielectric and mechanical properties in Ti_3_C_2_T_x_ MXene-MoS_2_/Methyl vinyl silicone rubber composites as flexible dielectric materials. J. Alloys Compd..

[44] Uddin A., Estevez D., Khatoon R., Qin F. (2023). Thermally Stable Silicone Elastomer Composites Based on MoS_2_@ Biomass-Derived Carbon with a High Dielectric Constant and Ultralow Loss for Flexible Microwave Electronics. ACS Appl. Mater. Interfaces.

[45] Singh S., Pal K. (2021). Investigation on microstructural, mechanical and damping properties of SiC/TiO_2_, SiC/Li_4_Ti_5_O_12_ reinforced Al matrix. Ceram. Int..

[46] Sethulekshmi A.S., Saritha A., Joseph K. (2022). A comprehensive review on the recent advancements in natural rubber nanocomposites. Int. J. Biol. Macromol..

[47] Deng H.T., Wen D.L., Feng T., Wang Y.L., Zhang X.R., Huang P., Zhang X.S. (2022). Silicone rubber based-conductive composites for stretchable “all-in-one” microsystems. ACS Appl. Mater. Interfaces.

[48] Jin L., Zhang C., Guo H., Wang H., Bai J., Zhao H. (2025). Improved electro-actuation of polydimethylsiloxane-based composite dielectric elastomer via constructing semi-interlocked dual-network. Polymer.

[49] Rius-Bartra J.M., Ferrer-Serrano N., Agulló N., Borrós S. (2023). High-consistency silicone rubber with reduced Young’s modulus. An industrial option to dielectric silicone rubber. J. Appl. Polym. Sci..

[50] Afolabi O.A., Ndou N. (2024). Synergy of Hybrid Fillers for Emerging Composite and Nanocomposite Materials—A Review. Polymers.

[51] Riaz A., Sarker M.R., Saad M.H.M., Mohamed R. (2021). Review on comparison of different energy storage technologies used in micro-energy harvesting, WSNs, low-cost microelectronic devices: Challenges and recommendations. Sensors.

[52] Zheng S., Guo H., Pan F., Meng F., Jiang H., Ruan L., Lu W. (2024). Breathable, durable, flexible, and battery-free full action response electronic textiles toward simply achieving the function of human skin. Nano Energy.

[53] Ouyang Y., Bai L., Tian H., Li X., Yuan F. (2022). Recent progress of thermal conductive ploymer composites: Al_2_O_3_ fillers, properties and applications. Compos. Part A Appl. Sci. Manuf..

[54] Abass B.A., Hunain M.B., Khudair J.M. (2021). Effects of Titanium Dioxide Nanoparticles on the Mechanical Strength of Epoxy Hybrid Composite Materials Reinforced with Unidirectional Carbon and Glass Fibers. IOP Conf. Ser. Mater. Sci. Eng..

[55] Lei R., Gao F., Yuan J., Jiang C., Fu X., Feng W., Liu P. (2022). Free layer-dependent piezoelectricity of oxygen-doped MoS_2_ for the enhanced piezocatalytic hydrogen evolution from pure water. Appl. Surf. Sci..

[56] Yun G., Tang S.Y., Lu H., Zhang S., Dickey M.D., Li W. (2021). Hybrid-filler stretchable conductive composites: From fabrication to application. Small Sci..

[57] Xu Q., Wen J., Qin Y. (2021). Development and outlook of high output piezoelectric nanogenerators. Nano Energy.

[58] Fu B., Li J., Jiang H., He X., Ma Y., Wang J., Hu C. (2022). Modulation of electric dipoles inside electrospun BaTiO_3_@ TiO_2_ core-shell nanofibers for enhanced piezo-photocatalytic degradation of organic pollutants. Nano Energy.

[59] Das T., Yadav M.K., Dev A., Kar M. (2024). Double perovskite-based wearable ternary nanocomposite piezoelectric nanogenerator for self-charging, human health monitoring and temperature sensor. Chem. Eng. J..

[60] Deng W., Zhou Y., Libanori A., Chen G., Yang W., Chen J. (2022). Piezoelectric nanogenerators for personalized healthcare. Chem. Soc. Rev..

[61] Nurazzi N.M., Asyraf M.R.M., Fatimah Athiyah S., Shazleen S.S., Rafiqah S.A., Harussani M.M., Khalina A. (2021). A review on mechanical performance of hybrid natural fiber polymer composites for structural applications. Polymers.

[62] Nasrin K., Sudharshan V., Subramani K., Sathish M. (2022). Insights into 2D/2D MXene heterostructures for improved synergy in structure toward next-generation supercapacitors: A review. Adv. Funct. Mater..

[63] Fan L., Yang L., Zhao D., Ma L., He C., He F., Zhao N. (2021). Balancing Strength and Ductility in Al Matrix Composites Reinforced by Few-Layered MoS_2_ through In-Situ Formation of Interfacial Al_12_Mo. Materials.

[64] Sezer N., Koç M. (2021). A comprehensive review on the state-of-the-art of piezoelectric energy harvesting. Nano Energy.

[65] Selleri G., Gino M.E., Brugo T.M., D’Anniballe R., Tabucol J., Focarete M.L., Zucchelli A. (2022). Self-sensing composite material based on piezoelectric nanofibers. Mater. Des..

[66] Wu N., Bao B., Wang Q. (2021). Review on engineering structural designs for efficient piezoelectric energy harvesting to obtain high power output. Eng. Struct..

[67] Zhang Y., Qi H., Sun S., Liu Y., Gao B., Wang L., Chen J. (2022). Ultrahigh piezoelectric performance benefiting from quasi-isotropic local polarization distribution in complex lead-based perovskite. Nano Energy.

[68] Han K.W., Kim J.N., Rajabi-Abhari A., Bui V.T., Kim J.S., Choi D., Oh I.K. (2021). Long-lasting and steady triboelectric energy harvesting from low-frequency irregular motions using escapement mechanism. Adv. Energy Mater..

[69] Dani S.S., Tripathy A., Alluri N.R., Balasubramaniam S., Ramadoss A. (2022). A critical review: The impact of electrical poling on the longitudinal piezoelectric strain coefficient. Mater. Adv..

[70] Zhao C., Benčan A., Bohnen M., Zhuo F., Ma X., Dražić G., Rödel J. (2024). Impact of stress-induced precipitate variant selection on anisotropic electrical properties of piezoceramics. Nat. Commun..

[71] Ali Z., Yaqoob S., Yu J., D’Amore A. (2024). Critical review on the characterization, preparation, and enhanced mechanical, thermal, and electrical properties of carbon nanotubes and their hybrid filler polymer composites for various applications. Compos. Part C Open Access.

[72] Ramesh M., Rajeshkumar L.N., Srinivasan N., Kumar D.V., Balaji D. (2022). Influence of filler material on properties of fiber-reinforced polymer composites: A review. E-Polymers.

[73] Nagaraja S., Anand P.B., Ammarullah M.I. (2024). Influence of fly ash filler on the mechanical properties and water absorption behaviour of epoxy polymer composites reinforced with pineapple leaf fibre for biomedical applications. RSC Adv..

[74] Liang T., Yuan Y.J. (2016). Wearable medical monitoring systems based on wireless networks: A review. IEEE Sens. J..

